# Identifying Antioxidant Proteins by Using Amino Acid Composition and Protein-Protein Interactions

**DOI:** 10.3389/fcell.2020.591487

**Published:** 2020-10-29

**Authors:** Yixiao Zhai, Yu Chen, Zhixia Teng, Yuming Zhao

**Affiliations:** Information and Computer Engineering College, Northeast Forestry University, Harbin, China

**Keywords:** antioxidant protein, unbalanced dataset, random forest, machine learning, sequence feature

## Abstract

Excessive oxidative stress responses can threaten our health, and thus it is essential to produce antioxidant proteins to regulate the body’s oxidative responses. The low number of antioxidant proteins makes it difficult to extract their representative features. Our experimental method did not use structural information but instead studied antioxidant proteins from a sequenced perspective while focusing on the impact of data imbalance on sensitivity, thus greatly improving the model’s sensitivity for antioxidant protein recognition. We developed a method based on the Composition of k-spaced Amino Acid Pairs (CKSAAP) and the Conjoint Triad (CT) features derived from the amino acid composition and protein-protein interactions. SMOTE and the Max-Relevance-Max-Distance algorithm (MRMD) were utilized to unbalance the training data and select the optimal feature subset, respectively. The test set used 10-fold crossing validation and a random forest algorithm for classification according to the selected feature subset. The sensitivity was 0.792, the specificity was 0.808, and the average accuracy was 0.8.

## Introduction

Reactive oxygen species (ROS) are products of metabolic processes (Birben et al., 2012) and include singlet oxygen, hydrogen peroxide, nitric oxide, superoxide anion radicals, and hydroxyl radicals. Excessive concentrations of ROS produce excessive oxygen radicals, and the antioxidant system in the organism cannot eliminate the ROS quickly enough, which causes oxidative stress (OS) (Schieber and Chandel, 2014). An excessive OS response can affect the destruction of the macromolecular structure, such as the DNA, proteins and other carbohydrates, and even give rise to cell death, which can lead to aging (Liguori et al., 2018) and initiate genetic diseases. At present, people have realized that the OS response has a role in the pathogenesis of many diseases, including cancer, acute and chronic kidney diseases, neurodegenerative diseases, cardiovascular disease, diabetes, and atherosclerosis (Pisoschi and Pop, 2015; Liguori et al., 2018).

In order to prevent excessively high concentrations of ROS from causing cell damage, the antioxidant proteins must be employed to strike a good balance between the oxidation process and the antioxidant process, which is essential. Given that antioxidant proteins have such powerful functions, accurate identification of antioxidant proteins is absolutely critical for revealing the deterioration of tissue function caused by certain diseases and aging, and for developing new types of antioxidant drugs that can treat or mitigate these types of diseases. However, traditional methods for identifying antioxidant proteins have the problems of being time-consuming and costly, such as western blots (Mahmood and Yang, 2012).

With the continuous improvement of genomic data (Xu et al., 2017; Wang et al., 2018; Zhou et al., 2018; Guo and Zou, 2019; Wang J. et al., 2020), sequencing technology and computer technology, data mining and machine learning methods (Quan et al., 2017; Zou et al., 2017) are being exploited to identify antioxidant proteins, and many researchers have already done so. In Feng et al. (2013) proposed an idea using Naive Bayes, based on sequence information, and after 3 years, they changed the method of data processing and proposed a model called AodPred (Feng et al., 2016). It was based on a support vector machine with 3-spaced residue pairs and its accuracy was significantly better than the former model. In 2016, an integration method was proposed by Zhang et al. (2016), which was applied for predicting antioxidant proteins with mixed features, indicating that protein secondary structure information facilitates the discrimination of target proteins. Then, a method called SeqSVM was presented by Xu et al. (2018) employing a 188D feature extraction method. Last year, Meng et al. (2019) also utilized a support vector machine with structural features to establish a model to discriminate target proteins.

Despite the strengths of the existing methods, there are still some shortcomings that have not been fully addressed. (1) Most methods did not consider the impact of data unbalances on classification when training samples. The feature subset after feature selection was more representative of the larger number of type (non-antioxidant proteins), and what we require to find is a feature subset that is more representative of antioxidant proteins. For example, in Meng’s experiment, the sensitivity and specificity of the test set results were 0.68 and 0.985, which meant that the sorted features were more conducive to the selection of non-antioxidant proteins. These problems also existed in Xu’s research, even if she did use an unbalanced treatment. (2) Features of protein secondary structure information are extracted based on the secondary structure predicted by sequence information using tools such as PSI-PRED (McGuffin et al., 2000). The whole process is complicated and time-consuming. In addition, there are errors in the predicted protein secondary structures, which also affect the accuracy of the features.

**FIGURE 1:**
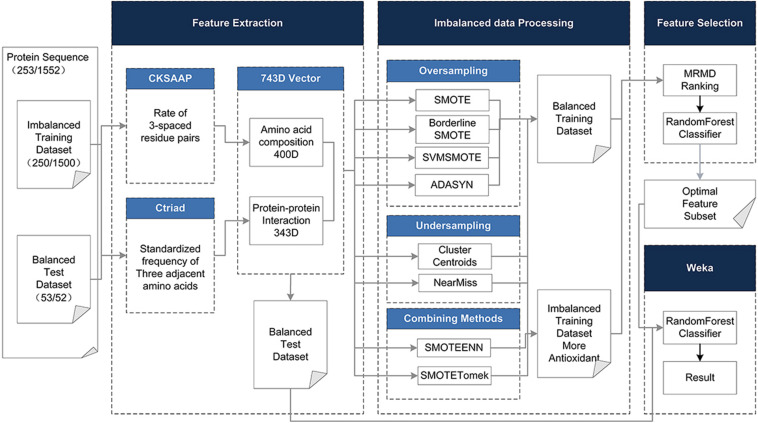
The method flowchart. The original dataset (training and test dataset) is processed in four phases. (1) Using CKSAAP and CT to extract 743D features. (2) In the unbalanced data processing phase, eight methods are adopted to balance the training dataset. (3) In the feature selection phase, the 743D features by MRMD score are ranked and the optimal feature set is selected by Random Forest classifier. (4) Use the selected feature subset to classify the test set to get the final result.

To address the above limitations and to enhance the predictive performance of the antioxidant proteins, the protein was described based on its hybrid features without structural information, including the Composition of k-spaced Amino Acid Pairs (CSKAAP) and the Conjoint Triad (CT) features. At the same time, taking into account the unbalanced state of the data volume of antioxidant proteins and non-antioxidant proteins, oversampling, under-sampling, and combined methods were used to process the dataset. The Max-Relevance-Max-Distance algorithm (MRMD) (Zou et al., 2016) could be exploited to single out the best feature subset for reducing the computational complexity and noise. On the contrary, we chose a 10-fold crossing test and random forest as the classifier, which has the characteristics of a fast running speed and less overfitting, rather than the very popular support vector machine. Figure 1 shows the complete data processing approach.

## Materials and Methods

### Benchmark Dataset

The dataset we used has been previously used by Feng et al. (2016), Xu et al. (2018), and Meng et al. (2019). We first collected proteins with antioxidant activities from the UniProt database (release 2014_02) according to the following steps: (1) only proteins with experimentally proven antioxidant activities were selected; and (2) ambiguous proteins were excluded, such as those containing non-standard letters like “B,” “X,” and “Z.” After this rigorous screening, we obtained 710 protein sequences as the original positive samples for the experiment. The negative samples were 1567 PDB proteins with identification values <20%, which were picked by PISCES-culled. To reduce redundancy and to avoid homology bias (Zou et al., 2020), peptides with more than 60% sequence similarity to each other were removed from the benchmark dataset by the CD-HIT program. Finally, the new dataset, including 1805 proteins sequences, was obtained, and 253 were antioxidant proteins and 1552 were non-antioxidant proteins. This can be expressed as follows:

(1) $$D\,a\,t\,a\,s\,e\,t=D\,a\,t\,a\,s\,e\,t_{+}\,\cup D\,a\,t\,a\,s\,e\,t_{-}$$

Where Dataset_+_ indicates the positive dataset, which contains 253 antioxidant proteins; Dataset_–_ indicates 1552 non-antioxidant proteins as the negative dataset; and the “∪” represents the symbol of “union” in the set theory, which means the benchmark dataset consisted of Dataset_+_ and *Dataset*_*–*_. The proportion of antioxidant to non-antioxidant samples is ∼1:6, which shows this is an unbalanced dataset.

As we all know, an unbalanced number of positive and negative samples will affect the accuracy. In order to prevent this from happening and to enhance the precision, 200 antioxidant and 1500 non-antioxidant proteins from the final benchmark dataset were selected as the training dataset and the rest with 53 antioxidant proteins and 52 non-antioxidant proteins was set as an independent testing dataset. The next section will detail how we deal with unbalanced training sets.

### Feature Extraction

The secondary structure information of the protein takes a long time to extract and process, and the calculation is complicated. In order to simplify the process, and at the same time, considering the diversity and complexity of the function of the antioxidant protein itself, mixture features were adopted to represent antioxidant proteins, including CKSAAP and CT. The CKSAAP describes the composition of amino acids. The other is a feature that describes protein-protein interaction (PPI) information (Yu et al., 2010, 2020; Liu et al., 2019b; Zhao et al., 2020). Three adjacent amino acids are regarded as a linker to judge the charge properties and hydrophobicity of the target protein. The iFeature was employed to extract features, which is a python toolkit. Assuming that a protein sequence consists of N amino acids, where *A*_*i*_ is the *i*th amino acid in the sequence, it can be defined as:

(2) $$P=A_{1}\,A_{2}\,A_{3},\cdots ,A_{N}$$

#### Composition of k-Spaced Amino Acid Pairs

The Composition of k-spaced Amino Acid Pairs (CKSAAP) feature delegates the component of amino acids (Tan et al., 2019; Liu et al., 2020). It calculates on behalf of the frequency of two amino acids separated by k residues (Chen et al., 2007a,b, 2008, 2009). Feng et al. (2016) has confirmed that a 3-spaced residue pairs feature is beneficial for classifying antioxidant proteins, and thus we only chose k=3 in our research, which picked up 400 dimensions. The 20 kinds of amino acids are combined in pairs to get 400 amino acid pairs. We can count the frequency of 400 amino acid pairs in a protein sequence. Then, a 3-spaced feature vector can be defined as:

(3) $$C\,K\,S\,A\,A\,P={\left[f_{1},f_{2},f_{3},\cdots ,f_{400}\right]}^{T}$$

where the T is the transpose of the *CKSAAP* vector and *f*_*i*_ is the frequency of the ith amino acid pair, which is defined as:

(4) $$f_{i}=\frac{n_{i}}{N-4}$$

where *n*_*i*_ is the number of times the ith amino acid pair appears in a protein sequence and *N* is the length of the sequence. The value of *N-4* represents the number of 3-spaced amino acid pairs in the whole protein sequence.

#### Conjoint Triad

**FIGURE 2:**
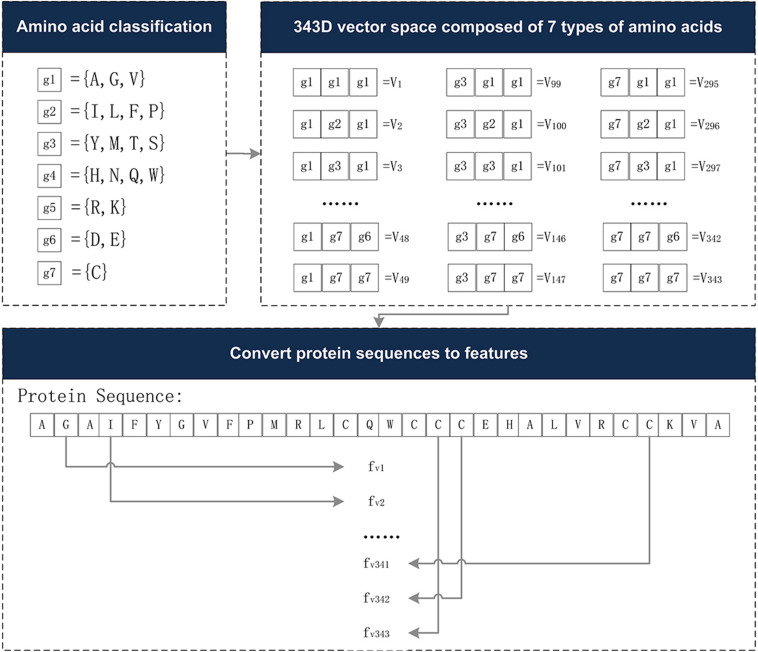
The 343-dimensional feature composition diagram. (1) Classify 20 amino acids into seven categories and obtain the g1∼g7, as shown in the amino acid classification part. (2) In the 343D vector space composed of seven types of amino acids, the seven amino acids were arranged and combined, resulting in 343 trimmers. (3) Examples of sequence conversion into features. The figure was adapted from the Supplementary Figure in Shen et al. (2007).

The Conjoint Triad descriptor (CT) describes the important information of protein-protein interactions (PPI). It is based on the triplet formed between amino acids and adjacent amino acids as the basic unit, considering the connections among them (Shen et al., 2007). First, by measuring the size and side chain volume of each amino acid dipole, and the effect of synonymous mutations, it classifies the 20 amino acids into seven categories. See Table 1 for the classification results of the 20 amino acids. According to the classification results and the three adjacent amino acids as a unit of this extraction method, we can use the CT algorithm to extract 343 dimensional features. The detailed definitions and descriptions for the structure of the 343 dimensional features are illustrated in Figure 2. Thus, the CT feature vector can be defined as:

**TABLE 1:** Classification of amino acids.

**No.**	***Dipolescale*^*a*^**	***Volumescale*^*b*^**	**Class**
1	−	−	Ala, Gly, Val
2	−	+	Ile, Leu, Phe, Pro
3	+	+	Tyr, Met, Thr, Ser
4	+ +	+	His, Asn, Gln, Tpr
5	+ + +	+	Arg, Lys
6	++′+′′	+	Asp, Glu
7	+ ^*c*^	+	Cys

*^*a*^*Dipole*   *scale* (Debye): −, Dipole < 1.0; +, 1.0 < Dipole <2.0; + +, 2.0 < Dipole < 3.0; + + +, Dipole > 3.0; ++′+′′, Dipole > 3.0 with opposite orientation. ^*b*^*Volume*   *scale* (*Å^3^*): −, Volume < 50; +, Volume > 50. ^*c*^Cys is separated from class 3 because of its ability to form disulfide bonds.*

(5) $$C\,T={\left[d_{1},d_{2},d_{3},\cdots ,d_{343}\right]}^{T}$$

where the T is the transpose of *CT* vector and *d*_*i*_ is the normalized frequency of the *i*th amino acid triad, which is defined as:

(6) $$d_{i}=\frac{v_{i}-m\,i\,n\,\left\{v_{1},v_{2},\cdots ,v_{343}\right\}}{m\,a\,x\,\left\{v_{1},v_{2},\cdots ,v_{343}\right\}}$$

where *v*_*i*_ is considered to be the frequency of these different trimmers in the antioxidant protein sequence. Finally, the above features follow the order of CKSAPP followed by CT, thus forming a set *FeatureSet* of 743 features, which can be defined as:

(7) $$F\,e\,a\,t\,u\,r\,e\,S\,e\,t={\left[f_{1},f_{2},f_{3},\cdots ,f_{400},d_{1},d_{2},\cdots ,d_{343}\right]}^{T}$$

### Unbalanced Data Processing

The unbalanced sample size will cause over-fitting of the sample with a large proportion (Wan et al., 2017; Fdez-Glez et al., 2018; Chao et al., 2019; Cheng et al., 2019; Liu, 2019), that is to say, the prediction is biased toward a classification with a larger number of samples, which will reduce the applicability of the model. The processing method at the data level is sampling. Under-sampling, over-sampling, and combined methods are three common and widely used approaches.

In this work, eight different methods from an unbalanced-learning library (Lemaître et al., 2017) were adopted to deal with the unbalanced data. These eight methods include SMOTE, ADASYN, BorderlineSMOTE, SVMSMOTE, ClusterCentroids, NearMiss, SMOTEENN, and SMOTETomek, which cover the three standard methods described above.

The data sets processed by the above methods were subjected to the same subsequent experimental operations, so as to compare the results obtained by different processing methods, and to select a method that is more suitable for processing antioxidant proteins.

### Feature Selection

If the extracted features are directly input into the subsequent classifier without any processing, it is difficult to obtain the ideal results (Wang et al., 2010; Tang et al., 2016, 2018; Basith et al., 2019; Liu and Li, 2019; Manavalan et al., 2019a). Further screening of the features, which can better reflect the characteristics of antioxidant proteins, is necessary. In this study, the Max-Relevance-Max-Distance algorithm (MRMD) was used for noise reduction. It mainly completes two steps, calculating the contribution of each feature to the classification first, and then selecting the best feature subset.

In order to rank all features of the sample, we calculated the MRMD score of each feature, which consists of a relevant value and a distance value. The relevant value is expressed as the relationship between the features and sample categories, calculated using the Pearson correlation coefficient as follows:

(8) $$P\,C\,C\,\left(\vec{X},\vec{Y}\right)=\frac{\frac{1}{N-1}\,\sum_{k=1}^{N}\left(x_{k}-\bar{x}\right)\,\left(y_{k}-\bar{y}\right)}{\sqrt{\frac{1}{N-1}\,\sum_{k=1}^{N}{\left(x_{k}-\bar{x}\right)}^{2}}\,\sqrt{\frac{1}{N-1}\,\sum_{k=1}^{N}{\left(y_{k}-\bar{y}\right)}^{2}}}$$

*x*_*k*_ and *y*_*k*_ are the *k*th element of $\vec{X}$ and $\vec{Y}$, which are two vectors. $\bar{x}$ and $\bar{y}$ are, respectively, the mathematical expectations of $\vec{X}$ and $\vec{Y}$. And the value of MR (Max-Relevance) for every feature is defined as MR_*i*_. The $\vec{F_{i}}$ is a vector that is represented in the *i*th feature and $\vec{C}$ is a target class vector of each instance.

(9) $$MR_{i}=|PCC\left(\vec{F_{i}},\vec{C}\right)|\left(1\leq i\leq M\right)$$

The distance value measures the independence of every feature. The higher the distance, the greater the independence. The MRMD provides three methods for calculating distance. In our research, the choice is Euclidean distance. We utilized the Euclidean distance to calculate the distance between each feature $\vec{F_{i}}$ and the other features, which is defined as follows:

(10) $$ED\left(\vec{F_{i}},\vec{F_{k}}\right)=\sqrt{\sum_{k=1}^{N}{\left(x_{i}-x_{k}\right)}^{2}}\left(1\leq k\leq M,k\neq i\right)$$

Then, based on this formula, we can obtain the Euclidean distance value of each feature *MD*_*i*_, which is the final value of Max-Distance. The larger the *MD*_*i*_ value, the lower the redundancy.

(11) $$MD_{i}=\frac{1}{M-1}\sum ED\left(\vec{F_{i}},\vec{F_{k}}\right)\left(1\leq i\leq M\right)$$

According to *MR*_*i*_ and *MD*_*i*_, the MRMD score is defined as:

(12) $$M\,R\,M\,D=M\,R_{i}+M\,D_{i}$$

The features in the set are sorted from high to low according to the score of *MRMD*. Each time a feature with the highest *MRMD* score is added, the classifier of the random forest is input for sorting, and finally, the feature subset with the highest accuracy and the least feature number is selected.

### Random Forest

Random forest (Liaw and Wiener, 2002) is an integrated algorithm that integrates multiple trees through the idea of integrated learning. It has been widely used in bioinformatics (Liu et al., 2019a;Manavalan et al., b,c; Wang et al., 2019; Lv H. et al., 2020; Lv Z. B. et al., 2020; Wang M. et al., 2020). It is composed of N decision trees. After the sample is input into the random forest, each decision tree will get a classification result, then N trees will obtain N classification results. The voting results of all classification results are counted, and the category with the most votes is the final output.

In our study, we adopted the random forest as the classifier because it has several advantages suitable for our data. The feature dimension extracted by the combined method of CKSAAP and CT is very high. Even after dimensionality reduction, it still belongs to high-dimensional data. Random forest can handle high-dimensional data, and the accuracy rate is not affected. The training set is unbalanced, and the amount of data becomes larger after the oversampling method is used. Random forest processing is adopted, and the running speed is fast. It is particularly useful in estimating the inferred mapping, so that there is no need to debug many parameters like SVM (Huo et al., 2020).

### Measurements

In statistical prediction, there are three commonly used evaluation methods for checking the accuracy of the model (Wang et al., 2008; Basith et al., 2018, 2020; Liu et al., 2019c; Yu et al., 2019; Zhu et al., 2019; Hasan et al., 2020), including the independent dataset sampling test, the k-fold cross validation and the jack-knife test. The jack-knife test is a resampling technique that is suitable for estimating the deviation over the entire sample (Li et al., 2019; Yang et al., 2019). This method has also been used in previous studies, i.e., Feng et al. (2016) and Meng et al. (2019). However, in our study, the training dataset was balanced by oversampling and under-sampling. The training set and test set were mutually exclusive. In order to reduce the complexity of the calculation, 10-fold cross validation is employed. For binary classification problems, the commonly used evaluation indicators are sensitivity (Sn), specificity (Sp), accuracy (Acc), F-score (F), Matthew’s Correlation Coefficient (MCC), and the Area Under the Curve (AUC).

(13) $$S\,n=\frac{T\,P}{T\,P+F\,N}$$

(14) $$S\,p=\frac{T\,N}{T\,N+F\,P}$$

(15) $$A\,c\,c=\frac{T\,N+T\,P}{T\,P+F\,N+T\,N+F\,P}$$

(16) $$F=\frac{2\times T\,P}{2\,T\,P+F\,N+F\,P}$$

(17) $$M\,C\,C=\frac{T\,P*T\,N-F\,P*F\,N}{\sqrt{\left(T\,P+F\,N\right)\,\left(T\,P+F\,P\right)\,\left(T\,N+F\,P\right)\,\left(T\,N+F\,N\right)}}$$

where TP, FP, FN, and TN indicate true positive, false positive, false negative, and true negative, respectively. *F* is a weighted harmonic average of precision and recall, which can avoid the contradiction between both. MCC is suitable for measuring imbalanced data sets, which is an index used in machine learning to measure the classification performance of two categories. In addition, the AUC is an evaluation index that measures the pros and cons of the binary classification model, which can make a reasonable evaluation of the classifier when the samples are unbalanced (Zhao et al., 2015, 2017; Manavalan et al., 2018a,b; Yu and Gao, 2019; Tang et al., 2020). The larger the AUC value, the better the performance of the model. The value of AUC is the area enclosed by the receiver operating characteristic curve (ROC curve) and the *x*-axis and *y*-axis. The vertical axis and the horizontal axis of the ROC curve are Sn and (1-Sp).

## Results

### Comparison of the Different Feature Extraction Methods

According to existing research, it has been confirmed that a series of feature extraction methods are effective for classifying antioxidant proteins, such as g-gap dipeptides feature, CTD, SSI, RSA, PSSM, etc. Therefore, in the planning stage of the experiment, we chose CKSAAP and CTD, and CT combined separately without structure information, and looked for the most suitable feature combinations for the target protein. Among them, CKSAAP was divided into only containing 3-spaced residue pairs and containing *g*-spaced residue pairs (g=1,  2,  3,  4,  5).

In addition, we adopted the principle of a single variable, controlling other factors unchanged, only changing the method of feature extraction, and observed its impact on the experimental results. After the feature extraction was completed, SMOTE and MRMD were used to perform unbalanced processing and to select the optimal feature subset. The final result was obtained by using a random forest classifier and the 10-fold cross-validation method.

**FIGURE 3:**
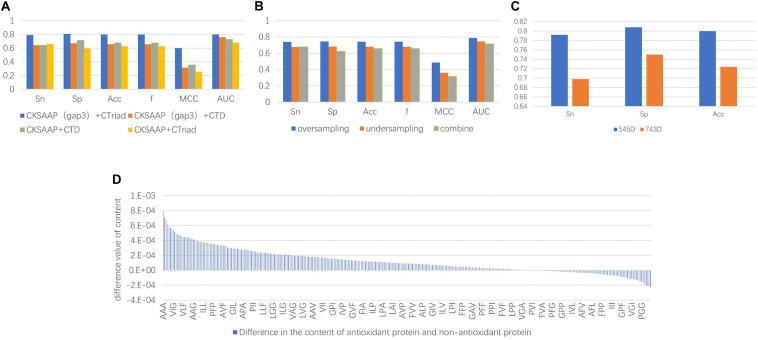
**(A)** The comparison chart of the results. It was obtained by different feature extraction methods and it shows that the results of the evaluation indicators using the CKSAAP+CT method were higher than the other methods. **(B)** The average value of the final results obtained by using three types of unbalanced processing methods. The result obtained by oversampling is much higher than the other methods. It can be seen that repeated sampling of a small number of sample data to synthesize new data is more conducive to extracting features that make it easy to distinguish antioxidant proteins. **(C)** Comparison of classification effects before and after dimensionality reduction with MRMD. Sn, Sp, and Acc are greatly improved, and the Sn and Sp results are very balanced. **(D)** Compared with sequence characteristics of the antioxidant proteins and non-antioxidant proteins, the triplet of the first type amino acid and the second type amino acid combination appears more frequently.

The experimental results indicated that the groups only containing 3-spaced residue pairs were superior than the others for classification, which also confirmed the conclusion that the 3-gap dipeptides feature in Feng et al. (2016) was good for classification. On the other hand, despite previous research showing that CTD could be used to obtain good classification results, such as the combined features of Zhang et al. (2016) and Xu et al. (2018) with 188D, in fact, the experimental results showed the classification accuracy of the CT groups was higher than that of the CTD groups. Therefore, only containing 3-spaced residue pairs and CT were selected as the methods of feature extraction. The comparison of the experimental results is shown in Figure 3A.

### Comparison of the Different Classifier and AodPred

In this experiment, three alternative classifiers were selected, namely LibSVM (Chang and Lin, 2011; Jiang et al., 2013), LibD3C (Lin et al., 2014), and Random Forest. LIBSVM is an SVM pattern recognition and regression software package, which was developed and designed by Prof. Lin Zhiren of Taiwan University. It has the characteristics of a simple to use method, fast operation speed and strong practicability. When using LibSVM, we input the training data into the gird.py file, and entered the values of the calculated parameters c and g into the LibSVM classifier embedded in WEKA (Hall et al., 2009), and then classified the test set. LibDC developed by Lin et al. (2014) is an integrated classifier, which combines multiple basic classification algorithms. Both LibD3C and Random Forest used the embedded WEKA version and we used their default methods to classify the test set.

The classification results showed that the two classification methods of LibSVM and LibD3C had the phenomenon of over-fitting, and the generalization ability of the test set was weak, while when using random forest, the generalization performance of the classification was stronger and more stable. Compared with the existing research AodPred, the method of random forest was higher than AodPred for sensitivity, specificity and accuracy. The comparisons of the experimental results are shown in Tables 2, 3. Table 2 shows the accuracies of 8 data unbalanced processing methods with different classifiers. Table 3 compares the best results in our research with the results of the known model AodPred. Our results were obtained after processing using the Smote method, dimensionality reduction using MRMD, and selected features using random forest classifiers applied to the test set.

**TABLE 2:** The accuracy rate of eight data imbalance processing methods in different classifiers.

	**RF**	**LIbD3C**	**LibSVM**
**Smote**	**0.8**	**0.571**	**0.533**
ADASYN	0.705	0.686	0.533
BorderlineSMOTE	0.733	0.638	0.533
SVMSMOTE	0.733	0.705	0.533
ClusterCentroids RandomState = 0	0.667	0.648	0.6
NearMiss version = 1	0.733	0.686	0.6
NearMiss version = 2	0.638	0.648	0.6
NearMiss version = 3	0.686	0.638	0.6
SMOTEENN	0.59	0.571	0.562
SMOTETomek	0.724	0.648	0.543

**TABLE 3:** Compared the best results in our research with the results of AodPred.

	**Sn**	**Sp**	**Acc**
AodPred	0.751	0.745	0.748
**Smote+RF**	**0.792**	**0.808**	**0.800**

### Comparison of the Different Unbalanced Data Processing Methods

We employed over-sampling, under-sampling and combined methods to deal with the unbalanced training data set. The methods used for oversampling were SMOTE, ADASYN, BorderlineSMOTE, and SVMSMOTE. The parameter settings of each method were the default parameters in the unbalanced library of python. The processed training set samples reached equilibrium, with 1500 positive examples and 1500 negative examples, respectively. ClusterCentroids and NearMiss were the methods of under-sampling. The parameter setting of ClusterCentroids was the default. The version parameters of the NearMiss method take 1, 2, and 3 for unbalanced data processing. Therefore, there were four actual undersampling methods. The processed training data contained 200 positive examples and 200 negative examples. SMOTEENN and SMOTETomek adopted SMOTE to combine with ENN and Tomek, respectively, which were combined methods. In our study, the parameter settings of both were also the default. After SMOTEENN, the processed dataset was also unbalanced, which including 1498 antioxidant proteins and 29 non-antioxidant proteins. Although the processed data was still in an unbalanced state, most of them were antioxidant proteins, which helped us screen out the features with obvious signals. Unlike SMOTEENN, the data processed by SMOTETomek was balanced, including 1500 positive examples and 1500 negative examples.

After the unbalanced training data, the optimal feature subset was selected by MRMD, and the test set was classified according to the different feature subsets. The experimental results showed that the model obtained by the data processed by the oversampling method had a higher sensitivity (Sn), specificity (Sp), accuracy (Acc), f score (F), Matthew’s Correlation Coefficient (MCC), and the Area Under the Curve (AUC) than the other two methods. The reason is that there are fewer antioxidant proteins, and repeated sampling of samples to strengthen their signal characteristics is more conducive to screening out antioxidant proteins. The comparisons of experimental results are shown in Table 4 and Figure 3B. Table 4 is the prediction results of the model established by different data unbalanced processing methods in the test set. Figure 3B shows the average of the prediction results of the models created by the three basic data unbalanced processing methods in the test set.

**TABLE 4:** The prediction result of the model established by different data imbalance processing methods.

	**Sn**	**Sp**	**Acc**	**F**	**MCC**	**AUC**
**SMOTE**	**0.792**	**0.808**	**0.8**	**0.8**	**0.6**	**0.8**
ADASYN	0.698	0.712	0.705	0.705	0.41	0.766
BorderlineSMOTE	0.736	0.731	0.733	0.733	0.467	0.807
SVMSMOTE	0.736	0.731	0.733	0.733	0.467	0.78
ClusterCentroids RandomState = 0	0.604	0.731	0.667	0.665	0.337	0.743
NearMiss version = 1	0.755	0.712	0.733	0.733	0.467	0.775
NearMiss version = 2	0.66	0.615	0.638	0.638	0.276	0.733
NearMiss version = 3	0.698	0.673	0.686	0.686	0.371	0.734
SMOTEENN	0.604	0.557	0.59	0.59	0.181	0.628
SMOTETomek	0.755	0.692	0.724	0.724	0.448	0.805

### Feature Contribution and Importance Analysis

The dimension of the original feature was 743D. After the feature selection of MRMD, the selected feature subset contained 545 features. Compared with the original features, the accuracy of the test set classification was improved by 0.076. The experimental results after dimensionality reduction were as follows: the sensitivity was 0.792, the specificity was 0.808, and the average accuracy was 0.8. Compared with the original method, the sensitivity was greatly improved. The comparison chart is shown in Figure 3C.

Not only that, by comparing the characteristic MRMD scores, we recognized that CT scores were generally higher than CKSAAP, and the characteristic scores composed of triplets composed of the first and second amino acids in CT were the highest. This means that there were differences in these characteristics between the positive and negative examples. Therefore, we counted the differences in the content of the triplet composed of the first type (A, G, V) and second type (I, L, F, P) of amino acids. There were a total of 343 triplets composed of these amino acids. Among the 260 features, the average content in antioxidant proteins was higher than that of non-antioxidant proteins. The content difference chart is shown in Figure 3D. A, V, I, L, F, and P were hydrophobic amino acids, and the tripeptide group composed of them was also hydrophobic, and thus we can infer that the hydrophobicity of proteins can be used to classify antioxidant proteins.

## Discussion

In this paper, we proposed a method with CKSAAP and CT features to identify antioxidant proteins. SMOTE was adopted to deal with unbalanced data, and we selected the optional feature set with MRMD. Using the 10-fold cross-validation and random forest classifier on the test set, we obtained an average accuracy of 0.8. The sensitivity and specificity were 0.792 and 0.808, respectively. We revealed that due to the small number of antioxidant proteins, when dealing with an unbalanced problem, oversampling to strengthen the antioxidant proteins makes it easier to discover the signal characteristics that represent the proteins. Therefore, oversampling is more suitable than under-sampling and combination methods. From the experimental results, the SMOTE method works the best. Additionally, after analyzing the characteristics, we found that the sequence of the antioxidant protein is more obvious in the triplets composed of hydrophobic amino acids, so we infer that the hydrophobicity of the protein can be used to classify the antioxidant proteins.

## Data Availability Statement

Publicly available datasets were analyzed in this study. This data can be found here: https://github.com/MAX-zyx/antioxidant_dataset.git.

## Author Contributions

YMZ conceived and designed the project. YXZ and YC conducted the experiments and analyzed the data. YXZ and YMZ wrote the manuscript. ZXT and YMZ revised the manuscript. All authors read and approved the final manuscript.

## Conflict of Interest

The authors declare that the research was conducted in the absence of any commercial or financial relationships that could be construed as a potential conflict of interest.
